# Does caffeine ingestion affect the lower-body post-activation performance enhancement in female volleyball players?

**DOI:** 10.1186/s13102-022-00488-0

**Published:** 2022-05-25

**Authors:** Aleksandra Filip-Stachnik, Michał Spieszny, Lidia Stanisz, Michał Krzysztofik

**Affiliations:** 1 Institute of Sport Sciences, The Jerzy Kukuczka Academy of Physical Education in Katowice, Mikołowska 72A str., 40-065 Katowice, Poland; 2 Institute of Sports Sciences, University of Physical Education in Krakow, Kraków, Poland; 3 Faculty of Physical Education and Sports, University of Physical Education, Kraków, Poland

**Keywords:** Post-activation potentiation, Power output, Resistance training, PAP, PAPE, Complex training

## Abstract

**Background:**

Post-activation performance enhancement (PAPE) is a physiological phenomenon that acutely improves voluntary muscular performance following a conditioning activity. A large and growing body of literature has investigated different strategies to induce a PAPE stimulus; however, little attention has been given to whether acute caffeine ingestion could augment the effect of PAPE on subsequent performance. This study evaluated the acute effects of caffeine ingestion and back squat conditioning activity on subsequent countermovement jump (CMJ) performance in female semi-professional volleyball players.

**Methods:**

Fourteen resistance-trained female volleyball players (26 ± 3 years) performed 3 different testing conditions in randomized order: where each ingested 6 mg/kg of caffeine (CAF) or placebo (PLAC) and performed a single set of back squats at 80%1RM until mean movement velocity dropped by 10% as the conditioning activity or a control (CTRL) condition where participants did not ingest any supplement and did not perform the conditioning activity. CMJ height was examined at baseline and in 2 min intervals until 10 min postconditioning activity. Furthermore, due to the wide inter-individual variation in optimal recovery time of PAPE response, the baseline and best post-conditioning activity performance were also analyzed.

**Results:**

The Friedman test revealed a significant difference in jump height within CTRL (*p* = 0.002) and CAF (*p* = 0.001) conditions, but no significant difference was found within the PAP condition. The post hoc showed a significant decrease in jump height in 8^th^ min in CTRL condition (*p* = 0.022, effect size [ES] = −0.31), a significant increase in jump height in 2^nd^ min in CAF condition (*p* = 0.013, ES = 0.3), without differences in PLAC condition in comparison to baseline values. Moreover, a significant jump height increases from baseline to best performance post conditioning activity value for CAF (*p* = 0.001, ES = 0.39) and PLAC (*p* = 0.001, ES = 0.3) condition, but no significant difference was found for the CTRL condition.

**Conclusions:**

The single set of heavy-loaded back squats with controlled velocity used as a conditioning activity in the current study enhanced subsequent CMJ performance in female volleyball players with no additional effect of caffeine.

## Introduction

Sports professionals and coaches are constantly seeking ways to improve physical fitness. Eagerly used and widely researched is the effect of post-activation performance enhancement (PAPE), which augments voluntary muscle performance [1, 2]. This phenomenon is achieved by using a conditioning activity before a subsequently performed similar explosive. Although the exact mechanisms underlying PAPE are still debatable, the increased muscle activation, temperature, and muscle water content were mentioned to date as those which might contribute to the reported performance enhancement [1, 3].

While PAPE alone offers an attractive solution for coaches to improve muscle performance, other means may be synergistic when combined with PAPE. Specifically, caffeine administration has enhanced explosive exercise performance [4–7]. Caffeine is similar to adenosine, and thus it influences central nervous system effects and modifies arousal, which may result in performance improvements [8]. Additionally, caffeine increases calcium release from the sarcoplasmic reticulum and motor unit recruitment [9]. At the same time, the benefits of PAPE have been documented for a wide range of sports-specific tasks [10–12]. Thus, the combined effects of PAPE and caffeine use may result in a more forceful muscular contraction and augment positive effects on explosive exercise performance. Unfortunately, only one study has so far analyzed that combination for acute performance enhancement [13]. The study by Guerra et al. [13] showed that a conditioning activity (complex of plyometric exercises and sled towing) combined with caffeine ingestion (5 mg/kg body mass) enhanced jumping performance to a greater degree than the same conditioning activity with placebo among male soccer players. Performance enhancement may occur due to the concomitant effects of post-activation induced increase in the rate of force development and caffeine-induced decrease in contraction time, as previously suggested [14]. However, no previous study assessed this combination in women. It is worth noticing that the results from a recently performed systematic review [15] showed differences in ergogenic effect of caffeine on resistance exercise between men in women, despite similar dosage and training levels.

Considering that the combined use of the PAPE protocol along with caffeine ingestion may be a promising strategy for acute sports performance enhancement, this study evaluated the acute effects of caffeine ingestion and back squat conditioning activity on subsequent countermovement jump (CMJ) performance. Since vertical jump performance has been reported as the most essential physical attribute directly related to game success [16] in volleyball, we decided to conduct this study on female semi-professional volleyball players. It was hypothesized that greater PAPE magnitude would be observed after the combined use of caffeine ingestion and the conditioning activity.

## Materials and methods

### Experimental design

**Fig. 1:**
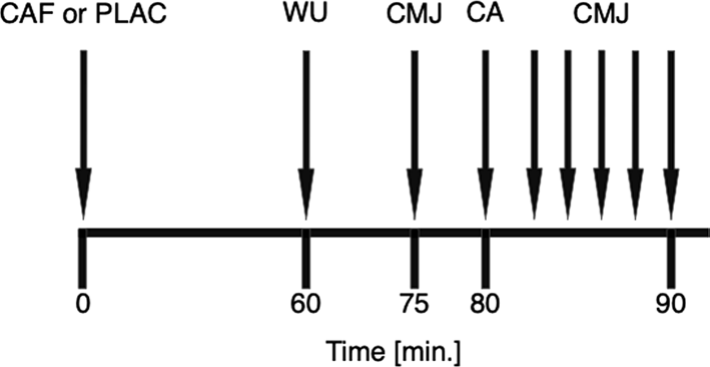
Experimental protocol design. CAF—caffeine ingestion; PLAC—placebo in-gestion; WU—warm-up; CMJ—countermovement jump assessment; CA—conditioning activity

The participants took part in a familiarization session and three randomized experimental sessions within 2 weeks, each separated by at least 72 h (Fig. 1). The familiarization session included the determination of the 1RM load for the back squat and 2 sets performed till 10% velocity-drop at 80%1RM. The experimental sessions were performed in randomized order, where each participant ingested 6 mg/kg of caffeine (CAF) or placebo (PLAC) or performed the exercise protocol without ingesting any substance (control, CTRL). During experimental trials, participants performed a single set of back squats at 80%1RM until mean movement velocity dropped by 10% as the conditioning activity (after ingestion of substance) or a control condition in which participants did not perform any conditioning activity and did not ingest any substance (after no supplementation). The use of velocity drop during conditioning activity allows for better optimization by taking into account individual fatigue assessment. Moreover, such settings have been previously found to be effective in inducing the PAPE effect [17, 18]. To assess changes in jump height, a single-set of 2 repetitions of the CMJ was performed before and after the conditioning activity in 5-time points with 2 min rest intervals. Both caffeine and the placebo (in the respective trials) were administered orally 60 min before the onset of the exercise protocol to allow peak blood caffeine concentration and at least 2 h after their last meal to avoid the influence of feeding on absorption rates [19]. Caffeine was provided in commercially available capsules (Caffeine Kick®, Olimp Laboratories, Dębica, Poland). The manufacturer of the caffeine capsules also prepared identical placebo capsules filled with an inert substance (all-purpose flour). We selected such a dosage of caffeine since it has consistently been shown to improve physical performance [20]. Participants were instructed to not perform any additional resistance exercises within 72-h of testing to avoid fatigue. Moreover, they were asked to maintain their nutrition, sleep and training habits throughout the study and not to use any additional supplements, caffeine sources and alcohol for 24-h before the sessions. Adherence to those instructions was verified prior to data collection. Habitual caffeine intake was evaluated by using a modified version of the validated questionnaire by Bühler et al. [19] and was assessed for the four weeks before the start of the experiment following previous recommendations [20].

### Participants

A sample size estimation using G*Power software (version 3.1.9.2, Dusseldorf, Germany) with the following variables parameters: “ANOVA, repeated measures, within factors” showed that to detect an effect size (ES) of g = 0.38 [21] would require 9 participants to provide 80% power with a significance level of 0.05 and correlation among repeated measures of 0.5 in this design for one group of participants, and three experimental conditions. Effect size of 0.38 was chosen given that this value was obtained from a meta-analysis investigating the effects of a heavy preconditioning activity on muscle power [21]. To account for potential drop-outs, we recruited fourteen resistance-trained female volleyball players who participated in the study (Table 1). The study participants had at least 2 years of resistance training experience and at least 7 years of volleyball training before enrollment in this study. The female players were allowed to withdraw from the experiment at any moment. The inclusion criteria were as follows: (i) free from neuromuscular and musculoskeletal disorders, (ii) resistance-trained, (iii) self-described satisfactory health status. Participants were excluded if they reported (i) a positive smoking status; (ii) a potential allergy to caffeine. They were informed about the objectives and potential risks of the study before providing their written informed consent for participation. The study protocol was approved by the Bioethics Committee for Scientific Research at the Academy of Physical Education in Katowice, Poland (3/2019) and performed according to the ethical standards of the Declaration of Helsinki, 2013.

**Table 1:** Descriptive characteristics of the study participants

Age [years]	26 ± 3
Body Mass [kg]	62.6 ± 5.6
Body Fat [%]	19.5 ± 3.9
Height [cm]	171 ± 5
Experience in resistance training [years]	6 ± 3
Experience in volleyball training [years]	13 ± 3
Relative back squat 1RM [kg]	1.48 ± 0.12
Habitual caffeine intake [mg/kg/b.m/day; mg/day]	2.9 ± 2.4 / 184.9 ± 153.3

1RM—one - repetition maximum

### Familiarization session and 1RM strength test

The participants arrived in the laboratory at the same time of day as the upcoming experimental sessions (in the evening between 5:00 and 7:00 pm). Firstly, the following anthropometric measurements were taken: height (WPT-60/150OW, Radwag, Poland), body mass and body fat percentage (InBody 370, Biospace Co., South Korea). A week before the main experiment, the 1RM back squat test was performed as described by Gepfert et al. [22]. Participants performed a standardized warm-up consisting of cycling on a stationary bike for 5 min (Keiser M3 Indoor Bike, Keiser Corporation, Fresno CA) at a resistance approximately of 100 W and cadence within 70–80 rpm; 2 circuits of 10 body-weight squats, 10 trunk rotations and side-bends; 10 internal, external and lateral arm swings; 5 split stance squats for each leg. Next, the participants performed 10, 6, 4, and 3 repetitions of the squat, respectively, starting at a load of 20 kg and progressing to 60–80% of their estimated 1RM. Then the load was gradually increased by 2.5–5 kg for each subsequent attempt until the participant was unable to perform a lift with proper technique according to the rules of the International Powerlifting Federation [23]. Participants were instructed to perform each repetition with a 2 s duration of the eccentric phase and maximal velocity in the concentric phase of the movement [24, 25]. The 1RM was defined as the highest load completed without any help from the spotters. Five-minute rest intervals were allowed between the 1RM attempts, and all 1RM values were obtained within five attempts. Following the 1RM test, all participants performed two sets of back squats till a 10% mean velocity-drop at 80%1RM.

### Experimental sessions

In a randomized order, after identical warm-up as before the 1RM test, the participants performed a control condition and 2 different experimental conditions, at least 72-h apart: (i) a control condition where participants did not ingest any capsules and did not perform the conditioning activity (CTRL), but only the CMJ, (ii) a caffeine condition (CAF) where each participant ingested 6 mg/kg of caffeine and performed a single set of back squats at 80%1RM, with repetitions performed until mean movement velocity dropped by 10% as the conditioning activity, (iii) a post-activation performance enhancement condition (PLAC) where each participant ingested placebo and performed the same conditioning activity as in CAF condition. To assess changes in jump height, single sets of 2 repetitions of the CMJ were performed. The CMJ was performed 5 min before and re-evaluated in the following time points: 2, 4, 6, 8, 10 min. The best repetition was kept for further analysis. Verbal questioning before data collection showed that players were unable to indicate correctly caffeine or placebo conditions (odds no greater than chance or 50:50).

### Measurement of movement velocity during the conditioning activity

A GymAware Powertool (Kinetic Performance Technology, Canberra, Australia) linear position transducer was used to assess the mean velocity-drop during the conditioning activity. The participants performed repetitions until the mean velocity dropped by 10% from the highest attained. This device provides reliable and valid data [26]. The external end of the cable was attached to the side of the bar and provided no resistance. The device was placed on the floor directly under the bar, with the magnetic bottom positioned on a weight plate to ensure no movement during each lift. The velocity of the barbell was recorded at 50 Hz.

### Measurement of countermovement jump performance with arm swing

The CMJ starting position was a standing position with a straight torso and knees fully extended with the feet shoulder-width apart, and hands were free to move. This type of jump was chosen because it more closely replicates the competitive conditions in volleyball. A previous study indicated excellent intersession reliability for CMJ arm swing jump height (intraclass correlation coefficient: 0.927) [27]. In the current study it was 0.989 (with a 95% confidence interval of 0.967 to 0.997); however, it has to be noted that it was measured on basis of CTRL and PLAC, therefore, the placebo effect couldn’t be excluded. Participants were instructed to perform a quick downward movement (approximately 90° of knee flexion) and, afterwards a fast-upward movement to jump as high as possible. If at any point the subject exhibited excessive knee flexion once air-borne, the jump was ruled invalid and repeated. The Optojump photoelectric cells (Microgate, Bolzano, Italy) device is an infrared platform with proven validity and reliability for assessing vertical jump height [28]. The device measures the flight of vertical jumps with a sampling frequency of 1000 Hz.

### Statistical analyses

Statistical analysis was performed using SPSS (version 25.0; SPSS, Inc., Chicago, IL, USA), and data were expressed as means with standard deviations (± SD). Moreover, the 95% confidence intervals for mean values and relative differences (i.e. in percentages) between baseline (BA) and postconditioning activity values were also calculated. Statistical significance was set at *p* < 0.05. The normality of data distribution was checked using Shapiro–Wilk tests. The differences in the highest attained mean velocity, and repetition performed until the mean velocity dropped during the CA performed in PLAC and CAF conditions were examined by paired sample t-tests since the data met normality assumptions. In contrast, the effects of the used CA on the jump height were examined by non-parametric statistics because Shapiro–Wilk test revealed a significant departure of data from normality. Related-samples Friedman’s ANOVA by ranks was used to examine for differences between all variable levels, and effect size (ES) was estimated by Kendall’s coefficient of concordance. When significant, pairwise comparisons were conducted using Wilcoxon signed-rank test with Bonferroni correction. Furthermore, since the optimal recovery duration shows a large inter-individual variability among athletes [17, 29, 30], additional analyses were made between the BA and the highest value obtained postconditioning activity irrespective of the rest interval in each trial. The magnitude of mean differences was expressed with standardized effect sizes; thresholds for qualitative descriptors of Hedges g were defined: ≤ 0.20 as “small”, 0.21–0.8 “medium”, and > 0.80 as “large” [31].

## Results

**Fig. 2:**
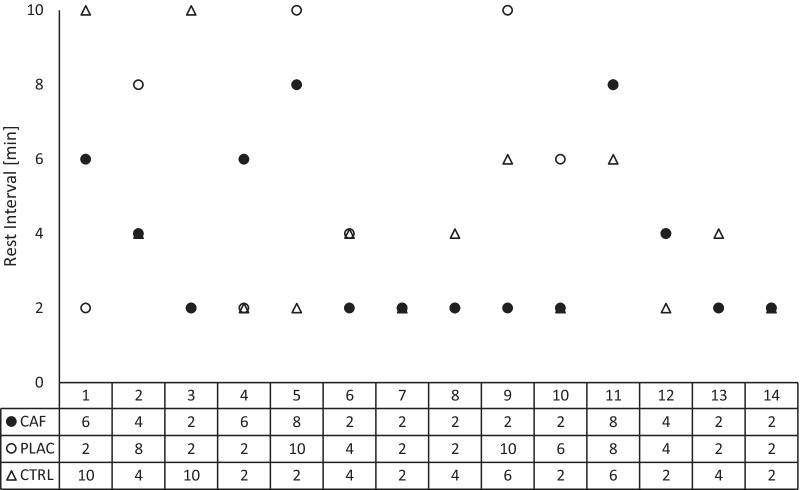
Individual rest intervals where the best performance postconditioning occurred

The paired sample *t*-test didn’t find a significant difference in mean velocity (0.62 ± 0.03 vs. 0.64 ± 0.02 m/s, *p* = 0.184; ES = 0.78) and repetition performed until the mean velocity drop between PLAC and CAF conditions (4.0 ± 1.2 vs 4.4 ± 1.4, *p* = 0.292; ES = 0.31) during CA. The Friedman test revealed a significant difference in jump height within CTRL (χ^2^(5) = 19.473; *p* = 0.002; Kendall’s W = 0.278) and CAF (χ^2^(5) = 19.875; *p* = 0.001; Kendall’s W = 0.284) condition, but no significant difference was found within the PAP condition. The post hoc showed a significant decrease at 8th min post conditioning activity (*p* = 0.022, ES = −0.31; Δ = −4.2 ± 4.5%) in comparison to the BA value for CTRL condition. Moreover, there was a significant increase in jump height in 2nd (*p* = 0.013, ES = 0.3; Δ = 3.83 ± 4.42%) min post conditioning activity in comparison to BA for the CAF condition (Table 2). The Friedman test for best performance post conditioning values revealed a significant difference in jump height between conditions in post conditioning activity values (χ^2^(2) = 9.926; *p* = 0.007; Kendall’s W = 0.354). Post-hoc testing showed a significantly higher jump height during the CAF condition in comparison to CTRL for best performance post conditioning activity values (*p* = 0.007, ES = 0.28; Δ = 3.7 ± 4.5%). Moreover, a significant jump height increases from BA to best performance post conditioning activity value for CAF (p = 0.001, ES = 0.39; Δ = 4.6 ± 4.1%) and PLAC (p = 0.001, ES = 0.3; Δ = 4.2 ± 3.4%) condition, but no significant difference was found for the CTRL condition (Table 2). Individual rest intervals where the best performance postconditioning occurred are presented in Fig. 2.

**Table 2:** Baseline and post conditioning activity countermovement jump performance

	BA (95 CI)	2 min rest (95 CI)	4 min rest (95 CI)	6 min rest (95 CI)	8 min rest (95 CI)	10 min rest (95 CI)	BEST (95 CI)
*Jump Height [cm]*							
CTRL	38 ± 4.3 (35.5 to 40.5)	37.8 ± 5 (34.9 to 40.7)	37.7 ± 4.8 (34.9 to 40.5)	37.1 ± 4.8 (34.4 to 39.9)	36.5 ± 5.2* (33.5 to 39.5)	37.2 ± 4.6 (34.5 to 39.9)	38.3 ± 4.7 (35.6 to 41)
PLAC	37.8 ± 4.3 (35.2 to 40.2)	38.6 ± 5.3 (35.6 to 41.7)	37.9 ± 5.1 (34.9 to 40.9)	38 ± 5.5 (34.8 to 41.2)	37.3 ± 5.2 (34.3 to 40.3)	37.8 ± 5.1 (34.9 to 40.7)	39.4 ± 5* (36.4 to 42.3)
CAF	37.9 ± 4.6 (35.3 to 40.6)	39.4 ± 5* (36.5 to 42.3)	39.0 ± 5.2# (36.0 to 41.9)	38.7 ± 5.2 (35.7 to 41.7)	38 ± 4.9 (35.2 to 40.8)	38.1 ± 5.3 (35.0 to 41.1)	39.7 ± 5.1*# (36.8 to 42.6)

Results are mean ± SD (95% confidence intervals)

*CAF* caffeine ingestion and application of conditioning activity; *PLAC* placebo ingestion and application of conditioning activity; *CTRL* control condition; *BEST* best performance post-conditioning activity irrespective of the rest interval

*Significant difference in comparison to the baseline within condition *p* < 0.05

^#^Significant difference in comparison to the corresponding time-point in CTRL *p* < 0.05

## Discussion

This study determined the effects of acute caffeine ingestion and a back squat conditioning activity on subsequent CMJ performance in semi-professional female volleyball players. The main findings were: (1) the heavy-loaded conditioning activity with velocity-control led to a significant, acute improvement of the subsequent CMJ performance, no matter if the caffeine was ingested or not; (2) after caffeine intake, the CMJ height increased at 2^nd^ minute of recovery time, while in the PLAC condition the significant increase was observed only when the best potentiated time was analyzed, which meant a wide inter-individual variation in recovery time; (3) the CMJ height following the conditioning activity after caffeine ingestion in comparison to placebo was comparable (~ 4.6% vs. ~ 4.2%), therefore no additional effect of caffeine was reported; (4) since there were no differences in jump height at baseline between CAF and PLAC conditions, this suggests that caffeine intake alone did not affect jump height. The novel finding of this study is that caffeine might modify the optimal recovery time of PAPE response. Thus, coaches and practitioners should consider when caffeine is administered before a conditioning activity.

Given that caffeine is a highly examined ergogenic aid, it's surprising that only one study so far has investigated its influence on the PAPE response [13]. The findings of this study are partially consistent with reports of Guerra et al. [13]. The authors also noted improvements in CMJ height after conditioning activity with (+ 5.75%) and without caffeine (+ 4.94%) ingestion. However, in contrast to the results obtained in this study, the magnitude of enhancement after conditioning activity combined with caffeine ingestion compared to placebo has reached the level of statistical significance. Interestingly, the individual optimal recovery time of PAPE responses was modified in the caffeine condition. After caffeine ingestion, the PAPE responses occur in the 2nd minute. This finding was not observed in a PLAC condition, where PAPE responses occurred highly individually. It is possible that caffeine had directly impacted the muscle tissue and increased motor unit recruitment, which resulted in a more forceful and faster muscular contraction [9, 32]. However, further investigations are needed to explore whether caffeine intake might result in earlier PAPE responses. Moreover, the results of the present study are in line with previous findings that it is essential to establish the individual response of each athlete, as the interpretation of the mean data is not always appropriate to determine the PAPE responses [17, 33–35].

It is worth noticing that differences in sex, training level, and habitual caffeine consumption between participants in this study and Guerra et al. [13] might explain the differences between the results of the current investigation. The effects of caffeine intake on CMJ performance in trained populations were previously assessed mainly among males [13, 36–40] or mixed sexes [41, 42], while only a few studies examined females [43–45]. Specifically, a systematic review [15] has suggested that the effects of caffeine during resistance exercise may be reduced in females compared to males ingesting the exact caffeine dosage. Furthermore, there is a reason to believe that the magnitude of PAPE may be sex-dependent due to the differences in muscle fibers proportions and strength levels between females and males. There are suggestions in the literature that athletes with a higher percentage of type II muscle fibers can exhibit a greater PAPE response [46], typically observed in males compared with females [47]. Additionally, the study by Rixon et al. [48] confirmed that differences in PAPE repones are sex and training experience-dependent. Therefore, further studies on the PAPE effect that directly compares females and males with caffeine ingestion are highly required.

Furthermore, CMJ height did not differ at baseline between CAF and PLAC conditions. This finding is contrary to the recent reports. A study by Norum et al. [44] indicated that caffeine's 4 mg/kg/b.m. significantly increased CMJ height (by 7.6 ± 4.0%) in resistance-trained females. In contrast, among professional female basketball players, Stojanovic et al. [45] found a small nonsignificant increase (by ~ 3.8 ± 7%) after 3 mg/kg/b.m. Several studies indicate that the ergogenic effect of caffeine may be greater in trained participants [8]; however, it seems that it wasn't the case here. Participants in the study by Norum et al. [44] showed only a slightly higher level of relative muscle strength in the squat (1.5 kg/b.m. vs. 1.48 kg/b.m.), while Stojanovic et al. [45] did not provide the strength level of the participants. However, based on the CMJ height, their fitness level was lower than that of the participants in this study. Thus, this does not seem to explain the lack of effect of caffeine ingestion. Also, the impact of habitual caffeine consumption does not seem to explain the lack of improvement in performance between caffeine conditions and others. Participants in the Stojanovic et al. [45] and Norum et al. [35] had higher habitual intake than the participants in our study (5.34 mg/kg/b.m. and 3.16 mg/kg/b.m. vs. 2.9 mg/kg/b.m.). However, both studies reported improvement after using even a lower dose of caffeine (3 and 4 mg/kg/b.m.). In contrast, participants in the study by Guerra et al. [13] had a lower habitual intake (1.39 mg/kg/b.m.). There were also no differences in the CMJ at baseline between caffeine and placebo. However, they noted a significantly greater PAPE effect in caffeine condition than in placebo, unlike in the current study. As a recent review suggested, it seems that the habitual caffeine intake did not mitigate its ergogenic impact [49].

Another matter that could have had a significant impact on the lack of improvements in CMJ performance at baseline and between the PAPE effect magnitude after caffeine may be the time of the day when the measurements were carried out. There is evidence that the caffeine ergogenic effect is mediated by ingestion time [4, 50]. In this study, all measurements were taken in the afternoon at the usual training time. On the contrary, Guerra et al.'s. [13], took all measurements in the morning. Stojanovic et al. [4] reported that caffeine ingested in the morning improved the vertical jump in basketball players, but not when participants took the same dose in the evening. This was also shown by Mora-Rodríguez et al. [50] by enhancement of propulsive velocity against different loads during bench press and squat exercises when caffeine was ingested in the morning. In contrast, the evening intake showed no improvements. Nevertheless, a finding that could potentially be relevant for athletes is that both CAF and PLAC effectively counteract fatigue, which was revealed in the 8^th^ minute following the conditioning activity in the CTRL condition. These results may have substantial applicability to many sports in which the capacity to repeat explosive bouts is essential. However, it has to be highlighted that caffeine intake modified the recovery time of PAPE response compared to the PLAC condition. It cannot be ruled out that this might be related to caffeine's small, albeit insignificant, effect on performance during CA as an increase in barbell velocity and the number of repetitions performed during CAF compared to the PLAC condition. Therefore, strength and conditioning coaches should keep in mind that the caffeine ingestion might affect the PAPE response and require an additional testing session.

In conclusion, the heavy-loaded conditioning activity with controlled velocity led to a significant, acute improvement of the subsequent CMJ performance, no matter if the caffeine was ingested or not. Although the observed differences between CAF and PLAC in combination with conditioning activity were not statistically significant, the caffeine intake modified the recovery time of PAPE response compared to PLAC. Moreover, there were no differences in the jump height at baseline between CAF and PLAC conditions, suggesting that caffeine intake alone did not affect jump height. However, the measurements were performed in the evening, probably contributing to the lack of caffeine's ergogenic effects.

## Conclusions

This study indicates that a heavy-loaded back squat with controlled velocity can be helpful in the acute enhancement of CMJ performance. Moreover, evening caffeine ingestion does not affect CMJ performance and has no additional impact to augment the effect of PAPE on subsequent performance. However, the strength and conditioning coaches should keep in mind that the caffeine ingestion might modify individual recovery time of PAPE responses. Therefore, it needs separate testing.

## Data Availability

The datasets analysed during the current study are available at https://doi.org/10.17632/swg8g8t6d3.1

## Abbreviations

PAPE: Post-activation performance enhancement
CMJ: Countermovement jump
CAF: Caffeine
PLAC: Placebo
1RM: One-repetition maximum
CTRL: Control
BA: Baseline
